# Real life evaluation of the multi-organ effects of Lumacaftor/Ivacaftor on F508del homozygous cystic fibrosis patients

**DOI:** 10.1186/s40360-022-00624-z

**Published:** 2022-10-20

**Authors:** Karin Yaacoby-Bianu, Zeev Schnapp, Ilana Koren, Anat Ilivitzki, Mohamed Khatib, Nadeem Shorbaji, Michal Shteinberg, Galit Livnat

**Affiliations:** 1 Pediatric Pulmonology Unit and CF Center, Carmel Medical Center, 7 Michal St, 3436212 Haifa, Israel; 2 B. Rappaport Faculty of Medicine, Technion – Israel Institute of Technology, Haifa, Israel; 3 Department of Pediatrics, Carmel Medical Center, Haifa, Israel; 4 Pediatric Endocrinology Unit, Carmel Medical Center, Haifa, Israel; 5 Pediatric Radiology Unit, Ruth Rappaport Children’s Hospital, Rambam Health Care Campus, Haifa, Israel; 6 Pulmonology Institute and CF Center, Carmel Medical Center, Haifa, Israel

**Keywords:** Cystic fibrosis, Lumacaftor-Ivacaftor, CFRD, F508del

## Abstract

**Background:**

Lumacaftor/Ivacaftor (LUM-IVA), a cystic fibrosis transmembrane conductance regulator (CFTR) protein corrector-potentiator combination, improves lung function and reduces pulmonary exacerbations (PEx) in F508del homozygous CF patients. However, the systemic effects of LUM-IVA outside the respiratory system have not yet been thoroughly investigated.

**Methods:**

A prospective, real-world, yearlong study was performed on F508del homozygous adult CF patients who commenced treatment with LUM-IVA. Pancreatic function, bone metabolism, fertility status, nutritional and pulmonary factors were evaluated.

**Results:**

Twelve patients, mean age 28.3 years (18.6–43.9) were recruited. Following 12 months of treatment, no changes were detected in glucose, insulin, c-peptide or BMI values. A significant relative decrease in mean alkaline-phosphatase levels (122.8 U/L vs 89.4, *p* = 0.002) and a trend toward an increase in calcium levels (9.5 vs 9.9 mg/dL, *p* = 0.074) were observed. A non-significant improvement in mean DEXA spine t-score after a year of treatment (-2.1 vs -1.6, *n* = 4, *p* = 0.11) was detected. Sweat chloride concentrations decreased significantly (-21.4 mEq/L; *p* = 0.003). Pulmonary outcome revealed improvement in spirometry values during the first three months (FEV_1_ by 5.7% *p* = 0.009, FEF_25-75_ by 4.3% *p* = 0.001) with no change in chest CT Bhalla score and CFQR after one year. There was also a significant decrease in parenteral antibiotic events (17 vs 8, *p* = 0.039) with shift from IV to oral antibiotics for PEx treatment.

**Conclusions:**

After one year of treatment, stabilization was observed in the pancreatic indices, nutritional status, structure and function of the lungs, with a beneficial effect on bone mineral metabolism and CFTR function. Additional studies should investigate the effect of CFTR modulators on extra-pulmonary manifestations.

**Supplementary Information:**

The online version contains supplementary material available at 10.1186/s40360-022-00624-z.

## Introduction

Cystic fibrosis (CF) is a genetic multisystem disease that is characterized by chronic airway infection, inflammation associated with loss of lung function, repeated pulmonary exacerbations (PEx), and ultimately, respiratory failure [1]. F508del is the most common mutation that causes CF. In July 2015, the U.S. Food and Drug Administration approved the combination Lumacaftor/Ivacaftor (Orkambi®, Vertex Pharmaceuticals) for use in patients with CF that are homozygous for the F508del mutation [2]. Briefly, this combination of a corrector (Lumacaftor) and potentiator (Ivacaftor) partially restores the activity of the membranous CF transmembrane conductance regulator (CFTR) protein. Lumacaftor improves the processing of F508del CFTR and its transport to the cell surface while Ivacaftor increases the probability for the channel to be open [2]. In clinical trials, treatment with Lumacaftor-Ivacaftor (LUM-IVA) led to increased lung function and weight gain, and a significant reduction in the frequency of PEx and CF-related hospitalizations [2–4].

Extra-pulmonary complications are common in CF. Of these, CF-related diabetes (CFRD) is one of the most severe prognostic factors [5] as there is a direct correlation between lung function and glycaemic control. In people with gating mutations responsive to Ivacaftor, treatment was associated with an improvement in insulin secretion after a glucose challenge [6]. Previous studies reported inconsistent effects of LUM/IVA treatment on glucose metabolism and acute insulin secretion [7–10].

Another important extra-pulmonary complication is CF bone disease (CFBD), characterized by low bone mineral density. Osteopenia and fractures are noted among 50–75% of patients with CF. These effects are attributed to malabsorption of fat-soluble vitamins, sex hormone deficiency, chronic infection and inflammation, and low levels of bone-building exercises resulting from advanced respiratory compromise, as well as primary CFTR dysfunction [11].

Delays in sexual maturity in CF patients secondary to their chronic disease are accompanied by low levels of luteinizing hormone (LH) and follicle-stimulating hormone (FSH) [12] with an increased risk of subfertility in women.

The current study aimed to evaluate the systemic effects of LUM-IVA treatment on endocrine pancreatic function, bone metabolism, and other extra-pulmonary parameters as well as respiratory changes during and after one year of treatment in adults with CF.

## Materials and methods

### Study design and participants

The study was a prospective, single center, observational study on F508del homozygous adults with CF who commenced treatment with LUM-IVA and were followed for one year. Pregnant or nursing women, solid organ or hematological transplant recipients, individuals with alcohol or substance abuse, patients with an acute upper or lower respiratory infection, and patients with PEx or changes in therapy within 28 days before Day 1 (first dose of LUM-IVA) were excluded.

All subjects received 400 mg Lumacaftor and 250 mg Ivacaftor (LUM-IVA) fixed-dose combination film coated tablets for oral administration every 12 h. All participants remained on their pre-study stable CF medication regimen throughout the year. They were followed in the CF Center at Carmel Medical Center, Haifa, Israel between November 2016 and June 2019. The institutional board reviewed and approved the study protocol, IRB number CMC108-16. All patients provided written informed consent prior to participation in the study. The ClinicalTrials.gov identifier of the study is NCT04623879.

In Israel, F508del allele frequency accounts for only around 23%, and therefore 13 adults with the F508del homozygous genotype attend our center.

### Study period

The screening period started on Day − 28 and ended on Day − 1. The treatment period started on Day 1 and lasted 12 months (± 7 days), with clinic visits scheduled every three months (Day 1 and Weeks 12, 24, 36, and 48 ± 7 days).

### Study assessments

The primary endpoint assessed pancreatic function via the absolute and relative change from baseline in oral glucose tolerance (OGTT) test through 12 months. Secondary endpoints included absolute and relative changes from baseline through 12 months in bone metabolism parameters, nutritional factors, reproductive hormones, sweat chloride, pulmonary status, and CF questionnaire-revised (CFQR) score.

#### Pancreatic function evaluation

At screening visit, 3 months, and 12 months, an OGTT was performed in patients without CF-related diabetes: 75 g of glucose were ingested, and glucose, insulin, and c-peptide were examined at three time points: 0, 1 h, and 2 h. In addition, HbA1C levels were evaluated in all patients at each study visit.

#### Bone indices

At screening and at 12 months, bone density was measured, using a dual-energy x-ray absorptiometry (DEXA) scan test. In addition, during every visit, bone metabolism factors, including parathyroid hormone (PTH), alkaline phosphatase, phosphorus, calcium, vitamin D levels (Vitamin D1-25-OH), and urine Ca/Cr ratio were assessed. All patients were treated with two DEKAs Plus soft gels every day (each soft gel containing 3000 IU vitamin D), as standard care in adult CF patients. Three patients received additional supplement of 2000 IU vitamin D every day, and one patient received 1000 IU every day. No patients received supplemental calcium.

#### Nutritional status

Body mass index (BMI) and levels of vitamin A, E (absolute), and albumin were assessed at each visit.

#### Fertility evaluation

Reproductive hormones including LH, FSH, testosterone, and estradiol were assessed at each visit in in both male and female participants.

#### Additional parameters

Vital signs, physical examination, sputum cultures, laboratory tests (e.g. complete blood count [CBC] and chemistry tests including electrolytes, liver and kidney function, and coagulation function) were assessed at every visit.

#### Pulmonary

Pulmonary and lung morphology evaluations were carried out by:

(1) The absolute change from baseline in the percentage of forced expiratory volume in one second (FEV_1_), forced vital capacity (FVC), and forced expiratory flow between 25–75 (FEF25-75), all were assessed at each visit. To obtain these parameters, spirometry was performed in accordance with the American Thoracic Society/European Respiratory Society (ATS/ERS) Task Force, using a KoKo® spirometer (n-Spire Healthcare, Inc., Longmont CO, USA) [13]. Absolute values of spirometry were transferred to percent predicted (pp) using Global Lung Function Initiative (GLI) reference data. (2) Chest computed tomography (CT) scans were performed at baseline and after one year, scored using the Bhalla scoring method [14] by a radiologist-investigator (the total score ranges from 9 to 25, with a higher score indicating more severe structural lung changes). (3) Quality of life was measured using the Cystic Fibrosis Questionnaire-Revised (CFQ-R) respiratory domain score each visit (scores range from 0 to 100, with higher scores indicating a better quality of life and four points considered to be a minimal clinically important difference) [15]. (4) PEx was defined as deteriorations in respiratory symptoms that led to changes in treatment [16]. Each PEx was considered a separate event, and the number of PEx during one year prior to commencing treatment with LUM-IVA was compared to PEx through the first year of treatment. We documented the number of exacerbations, oral vs. intravenous (IV) antibiotic treatments, hospitalizations, presence of fever > 38 °C, laboratory parameters: white blood cells count [WBC], absolute neutrophil count [ANC], and C-reactive protein [CRP] at the initiation of PEx (in hospitalized patients), sputum culture results, and time to next PEx.

#### CFTR function

Evaluation was measured through testing the concentration of sweat chloride that was performed at screening and after one year of treatment by Macroduct ® sweat collection system [17].

### Statistical analysis

Statistical analyses were performed using the SAS version 9.4 (SAS Institute, Cary North Carolina, USA). All measured variables and derived parameters were tabulated using descriptive statistics. For categorical variables, summary tables are provided presenting sample size and absolute and relative frequency. For continuous variables, summary tables are provided presenting sample size, arithmetic mean, standard deviation, median, minimum, and maximum for means of variables. Wilcoxon Signed-Rank test for paired samples was applied for testing the statistical significance of the changes from visits 3 and 12 to baseline in Table 2 and from visits 3, 6,9,12 to baseline in Tables 3 and 4, and Supplementary Table 1 and 2. A paired t-test for two means (repeated observations) was applied for testing the statistical significance of the change from baseline for each continuous variable. All tests were two-tailed, and a *p*-value of 5% or less was considered statistically significant.

## Results

### Baseline parameters

**Fig. 1:**
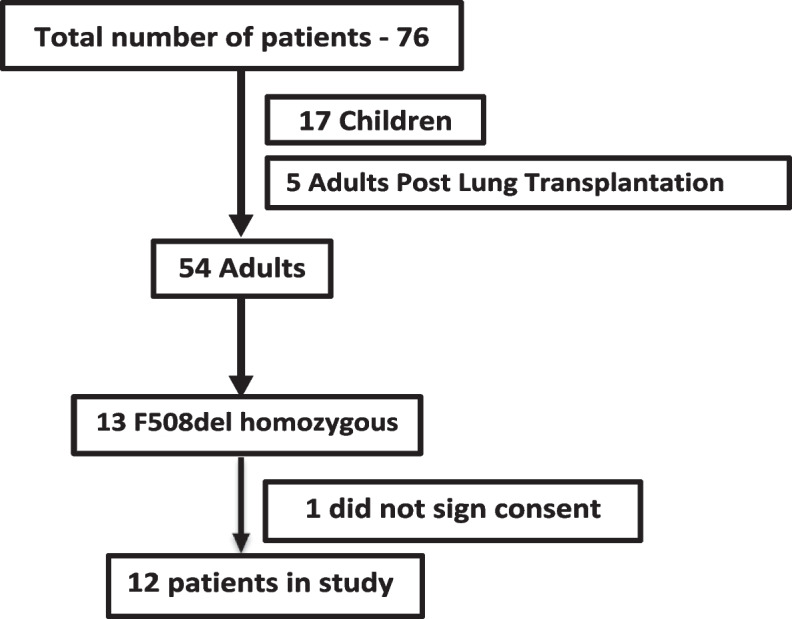
CF patient's flow chart

Out of 13 F508del homozygous adults attending Carmel CF Center who were screened, 12 consented (Fig. 1). 8 men (67%), mean age 28.3 ± 6.9 years and mean BMI 20.5 ± 3.4 kg/m^2^. All patients had pancreatic insufficiency (PI) and 4 patients had CFRD at initiation of treatment. Their baseline parameters are presented in Table 1. The values of pulmonary characteristics (ppFEV_1_ of 60 ± 16.9 and ppFEF_25-75_ of 30.1 ± 17.4) indicate a relatively progressed stage of CF lung disease at the start of treatment.

**Table 1:** Baseline characteristics of participants

Before Lumacaftor/Ivacaftor treatment (*N* = 12)	Mean (SD)
**Age in years at start**	28.3 (± 6.9)
**Male/ Female**	8 (67%) / 4 (33%)
**CFRD at start**	4 (33%)
**ppFEV1%**	60 (± 16.9)
**ppFVC %**	75.5 (± 16.7)
**ppFEF25-75%**	30.1 (± 17.4)
**BMI Kg/m**^**2**^	20.5 (± 3.4)
**Sweat chloride mEq/L** ^a^	107.7 (± 11.6)
**CT Bhalla score **^b^	12.8 (± 2.7)

^a^*N* = 11

^b^*N* = 10

### Endocrine pancreatic function assessments

In 8 patients (no CFRD) who underwent OGTT at screening, 3, and 12-month visits, no consistent changes were noticed in glucose, insulin, or c-peptide levels. Following one year of LUM-IVA treatment, the median glucose values at 2 h of OGTT decreased from 122 mmol/L (range 94–159) at screening to 109 mmol/L (range 84–215), without statistical significance (Table 2). No patients developed CFRD during the first year of LUM-IVA treatment. Also, none of the four CFRD patients were able to reduce the insulin dose under LUM-IVA treatment. The median value of HbA1C for CFRD patients was 9, and for non-CFRD 5.9, throughout the study with no detectable change (data not shown).

**Table 2:** Change in Oral Glucose tolerance test (OGTT) results from baseline

**Glucose tolerance test results**	**N**	**Mean**	**Std**	***P*-value of change from baseline** **Non parametric test** **Signed rank test**
**Baseline**^a^				
Insulin0	6	13.2	10.3	
Insulin1	6	81.8	84.5	
Insulin2	6	102.3	96.9	
Cpeptide0	6	0.3	0.1	
Cpeptide1	6	1.1	0.4	
Cpeptide2	6	1.6	0.5	
Glucose0	8	93.4	6.5	
Glucose1	7	151.3	25.3	
Glucose2	8	125.8	24.7	
**3 months after treatment**				
Insulin0	6	7.1	8.1	0.500
Insulin1	5	59	80	0.625
Insulin2	5	47.7	38	0.250
Cpeptide0	7	0.3	0	0.218
Cpeptide1	5	1.4	0.4	0.125
Cpeptide2	5	1.9	1.2	0.875
Glucose0	7	87.1	15.4	0.328
Glucose1	7	174.3	52.4	0.218
Glucose2	7	133.9	34.6	0.468
**12 months after treatment**				
Insulin0	8	6.1	9.8	0.375
Insulin1	8	35	58.7	0.125
Insulin2	8	28	42.8	0.125
Cpeptide0	8	134.5	378.8	0.812
Cpeptide1	8	164.7	463.2	0.437
Cpeptide2	7	38.2	98.7	0.125
Glucose0	8	98	20.2	0.718
Glucose1	8	197.9	47.5	0.193
Glucose2	8	124.4	41.9	0.109

^a^Baseline units: Insulin (mIU/L), C-peptide (nmol/L), Glucose (mg/dL)

### Bone metabolism assessments

**Fig. 2:**
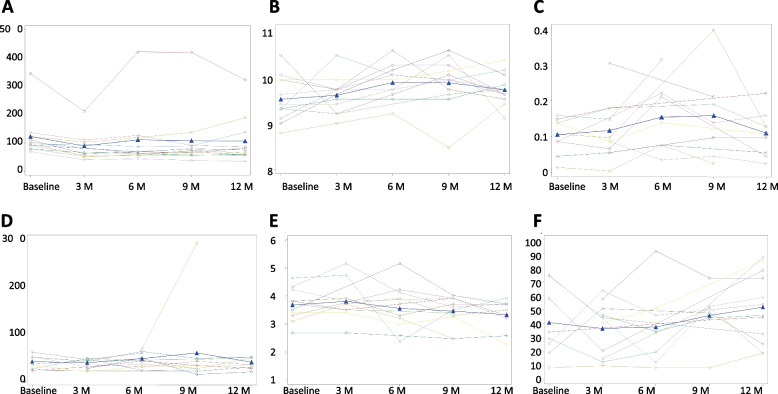
Bone metabolism parameters through 12 months of LUM-IVA treatment. Single subject and mean (blue solid) curves are presented for **A** Alkaline-Phosphatase (U/L), **B** Calcium (mg/dL), **C** CA/CR urine, **D** PTH (pg/mL), **E** Phosphor (mg/dL), **F** Vitamin D (nmol/L), X axis represents the study visits in months (M). Y axis represents a scale of the value of each tested parameter.

No deterioration was noted in any of the bone health parameters (Table 3, Fig. 2 A-F). A significant relative decrease in the mean alkaline-phosphatase level after 3 months of treatment (*p* = 0.002) and a trend towards increased calcium levels at 6 months of treatment were observed (Fig. 2 A, B). A non-significant concomitant increase in urinary excretion of calcium at 6 months was observed as well (*p* = 0.06) (Fig. 2 C). A trend towards an increase in Vitamin D levels was evident but did not reach statistical significance (Fig. 2 D). There was no change in phosphorus or PTH levels (Fig. 2 E, F). We observed a non-significant improvement in the mean spinal total T-score in DEXA test from -2.1 to -1.6, *p* = 0.11 in four patients who had DEXA tests at both the screening and 12-month visits. It is important to emphasize that none of the patients had previous pathological fractures.

**Table 3:** Mean values of bone parameters at baseline and after 3, 6, 9, and 12 months of Lumacaftor/Ivacaftor treatment

	**At Baseline**	**3 months after start**	**6 months after start**	**9 months after start**	**12 months after start**
**Vitamin D** (SD)	40.9 (± 24.5)	36.6 (± 19.2)	37.6 (± 25.9)	45.8 (± 18.5)	51.9 (± 24.3)
N	8	6	4	4	8
Change from baseline		-2.2	-12.6	3.6	9.2
*P*-value		1.00	0.750	0.750	0.382
**Phosphorus** (SD)	3.7 (± 0.6)	3.8 (± 0.7)	3.6 (± 0.8)	3.5 (± 0.5)	3.3 (± 0.5)
N	11	9	9	11	11
Change from baseline		0.1	-0.1	-0.2	-4.9
*P*-value		0.638	0.891	0.270	**0.022**
**Calcium** (SD)	9.5 (± 0.5)	9.6 (± 0.4)	9.9 (± 0.4)	9.8 (± 0.5)	9.7 (± 0.3)
N	12	12	11	12	12
Change from baseline		0.1	0.3	0.3	0.2
*P*-value		0.570	0.074	0.106	0.142
**PTH** (SD)	40.9 (± 14.4)	38.4(± 10.1)	47.1 (± 14.6)	59.2(± 81.1)	39.7 (± 11.6)
N	8	8	6	6	5
Change from baseline		-2.8	1.2	-2.4	5
*P*-value		0.546	1.00	0.843	0.250
**Alkaline-phosphatase** (SD)	122.8 (± 71)	89.4(± 49.2)	111.3(± 104)	108 (± 99.6)	106.8 (± 78.8)
N	12	10	11	12	12
Change from baseline		-37.5	-13.8	-14.8	-15.9
*P*-value		**0.002**	0.051	0.075	0.176
Relative change (%)		-28.1	-18.9	-18.8	-15.6
**Urine Ca/Cr** (SD)	0.1 (± 0.0)	0.1 (± 0.1)	0.2 (± 0.1)	0.2 (± 0.1)	0.1 (± 0.1)
N	9	8	6	4	7
Change from baseline		0	0.1	0.1	0
*P*-value		0.664	**0.062**	0.125	0.593
**DEXA: Spinal Total T-score** Mean (SD)	-2.1(± 0.5)				-1.6(± 0.4)
N	4				4
Change from baseline					0.5
*P*-value					0.110

### Nutrition, vitamins, liver, and fertility status

There was no impact on BMI values throughout one year of LUM-IVA treatment. However, a significant increase in albumin levels (Table 1 Supplementary) was found at the 6- and 9-month visits (4.4 vs 4.7 mmol/L *p* = 0.013 and 4.4 vs 4.7 mmol/L, *p* = 0.032, respectively). No noticeable changes were detected in the levels of liver enzymes (aspartate transaminase, alanine transaminase, and gamma glutamyl transferase), lipid-soluble vitamins (A, E), or fertility hormones (LH, FSH, testosterone, and estradiol) (data not shown).

### Cystic fibrosis transmembrane regulator activity

Sweat chloride test results indicated a decrease in chloride concentration after one year of treatment, at baseline mean 107.7 (± 11.6) vs 86 (± 12) mEq/L, *p* = 0.003 (change from baseline of -21 (± 15.3) mEq/L, *N* = 9).

### Pulmonary outcomes

Stable PFT values compared to baseline were observed after one year of treatment (ppFEV_1_ improved by 0.6% and ppFVC improved by 2.7% at 12 months) (Table 4). Moreover, at three months, a marked increase was evident (ppFEV_1_ by 5.7% *p* = 0.009, ppFEF_25-75_ by 4.3% *p* = 0.001) (Table 4). No change was observed in the chest CT Bhalla total score after one year of treatment (Table 4). No change in quality of life, as evaluated by the CFQR respiratory domain score, was noted (data not shown). Thirty PEx events were recorded in the year before starting LUM-IVA treatment as compared to 28 PEx during the study period. The mean PEx rate per patient year was 2.5 before LUM-IVA, and 2.3 during the study. There was no change in the number of hospitalizations nor in the duration of hospitalizations per PEx (Table 2 Supplementary). However, fewer PEx were treated with IV antibiotics (28.6% on LUM-IVA vs. 56.7% one year before *p* = 0.039) (Table 2 Supplementary). No difference was observed between the PEx events before and after LUM-IVA treatment with respect to time of the next PEx or complications such as hemoptysis, presence of fever, levels of WBC, ANC, and CRP at PEx start. Bacteriological assessments showed the presence of *Pseudomonas aeruginosa* (PA) in combination with methicillin susceptible *Staphylococcus Aureus* (MSSA) during PEx in most patients (data not shown).

**Table 4:** Mean respiratory values after Lumacaftor/Ivacaftor treatment

	**3 months** **after start**	**6 months** **after start**	**9 months** **after start**	**12 months** **after start**
	***N* = 12**	***N* = 11**	***N* = 12**	***N* = 12**
**FEV1%** (SD)	(16 ±)65.7	(18.3 ±)61.5	(17.6 ±)62.6	(15.8 ±)60.6
Change from baseline	5.7	0.7	2.6	0.6
*P*-value	**0.009**	0.861	0.34	0.921
**FVC%** (SD)	(18.6 ±)82.8	(17.7 ±)80.9	(18.2 ±)77.1	(17.5 ±)78.2
Change from baseline	7.3	4.6	1.6	2.7
*P*-value	**0.027**	**0.0371**	0.0775	0.0626
**FEF25-75%** (SD)	(17 ±)34.5	(17.2 ±)31.1	(17.3 ±)32.6	(19.9 ±)34
Change from baseline	4.3	0.5	2.5	3.9
*P*-value	**0.001**	0.764	0.181	0.672
**CT Score** (SD)^a^	**-**	**-**	**-**	(2.7 ±)12.5
Change from baseline	**-**	**-**	**-**	-0.3
*P*-value	**-**	**-**	**-**	0.517

^a^*N* = 10

## Discussion

This was a real-life prospective observational study evaluating the systemic effects of LUM-IVA on pancreatic endocrine function, bone metabolism, fertility status, nutritional-state, and pulmonary outcomes in 12 CF patients during a one-year period of treatment. The study population comprised of adult patients with a long-standing lung disease and a relatively high degree of pulmonary compromise. Left untreated, such patients tend to deteriorate over time, with an estimated annual decline of 1–2% in PFT in CF patients with the same genotype [2].

### Pancreatic function

No consistent changes in levels of glucose, insulin, or c-peptide were detected in this study. We found a trend towards a decrease in the 2-h glucose value after one year of LUM-IVA treatment, which is the most important time point for the decision to start insulin treatment. Previously reported findings on the impact of LUM-IVA treatment on the glucose levels are inconsistent, as some found no improvement in the glycemic control [7, 10], while others reported significant reductions in 1- and 2-h glucose levels [9]. Recently, Moheet et al. [8] evaluated 39 patients who had been prescribed LUM/IVA, and underwent OGTTs at baseline, and at 3, 6 and 12 months on therapy, and concluded, similar to our study, that LUM/IVA therapy did not improve insulin secretion or glucose tolerance. In addition, Kessler and colleagues [18] suggested that the CFTR modulators play a positive role at the very early stage of glucose tolerance abnormalities in CF, which is unfortunately not the case in our adult cohort.

### Bone metabolism

To the best of our knowledge, this is the first study evaluating the effects of LUM-IVA treatment on bone metabolism. We demonstrated decreases in alkaline-phosphatase and increases in calcium levels and urinary excretion of calcium. These changes may indicate improved vitamin D absorption, which mends the grade of osteomalacia and may potentially account for the lower alkaline-phosphatase levels [19]. These findings are in line with a case report [20] describing a 25 year old CF patient with osteomalacia which improved following a change in vitamin D levels. Similarly, a large CF-registry-data-based observational analysis of patients with a variety of gating mutations showed a lower prevalence and relative risk of CFBD in the Ivacaftor-treated group compared to controls [21]. In a small series by Sermet-Gaudelus and colleagues [22], involving 7 adults with the G551D mutation treated with IVA, a significant improvement in lumbar spine z-scores was observed. In our cohort, the tendency towards improved DEXA test results following one-year reflect improvement in factors contributing to osteopenia including CFTR dysfunction, malabsorption of fat-soluble vitamin D, and malnutrition [23].

### Nutritional status and fertility

Mean BMI did not change throughout the study period. This is in accordance with the results presented in real-life studies by Diab-Cáceres et al. [24] and Hubert, D et al. [25], but contrary to findings summarized in a systematic review of five randomized controlled trials [26] showing improved BMI, although their power for analysis was limited.

Reduced fertility in CF patients is common not only in men, but also in women, estimated at 35% [27]. We found no change in sex hormones following one year of treatment with LUM-IVA. Jones and colleagues [28], reported a series of female patients who previously required *in vitro* fertilization but were able to become pregnant spontaneously or to have normalized menstrual cycles after IVA treatment. To the best of our knowledge, no other studies examined fertility in CF patients treated with LUM-IVA.

### CFTR function

The mean decrease of 21.4 mEq/L in sweat chloride concentration after one year of treatment is numerically similar to that reported in a post-marketing authorization study by Graeber and colleagues [29] that assessed adults and children aged > 12 years. They demonstrated a mean reduction of 17.8 mEq/L in sweat chloride levels after 8–16 weeks of treatment [29].

### Pulmonary outcomes

Pulmonary function results suggest that the treatment prevented deterioration and could potentially, in the long term, delay the need for lung transplantation. The improvement in ppFEV_1_ of 5.7% and in ppFEF_25-75_ of 4.3% at three months of treatment was similar to the modest yet significant results in Phase III studies [2]. Our results were also in line with the PROGRESS study [4] in which there was a 42% slower rate of FEV_1_% decline over the study period. Recently, Sagel et al. [30] conducted a real-world multicenter study on a cohort of 193 children and adults, and reported no statistically significant or clinically meaningful changes in lung function and hospitalization rates for pulmonary exacerbations throughout a year of LUM/IVA treatment, resembling our real life results.

To the best of our knowledge, this study is the first to evaluate chest CT before and after a year of LUM-IVA treatment. Similar to PFTs, analysis of the CT Bhalla score suggested a lack of deterioration in lung structure. This is contrary to results of the CORK study [31], which evaluated patients with the G551D gating mutation after initiation of Ivacaftor and revealed an improvement in CT imaging scores. In the CORK study, investigators analyzed adult patients with a better baseline ppFEV_1_ than our cohort, with a relatively milder class 3 CFTR mutation (G511D). The lack of improvement observed in our cohort may be due to the already advanced level of lung morphology damage at baseline.

In our small cohort, no change was detected in quality of life, nor in a reduced rate of PEx or hospitalizations, contrary to previously reported studies [2, 3, 26, 32]. This may have resulted from one patient acquiring infection with *M. abscessus* during the study period. We interpret the shift from IV to oral antibiotics in treating PEx as reflecting a milder severity of PEx in our cohort. However, measures of inflammatory response such as CRP, blood leucocytes, and ANC were not affected.

During this study, patients did not experience any adverse events. None of the patients discontinued treatment.

This study had several limitations. First and foremost is the small sample size and the fact that it was conducted within a single center. Due to the small patient number and insufficient statistical power of the study, there are some insignificant results which represent a type 2 statistical error. The design of a real-world study has the inherent problems of data not being consistently available for all patients at all time-points. There was also variability and heterogeneity in clinical response to LUM-IVA treatment resulting from objective “real-life” reasons such as severe lung disease at baseline with ppFEV_1_ lower than 40%, new onset *M. abscessus* infection during the study period, and others.

In conclusion, this study on adult patients demonstrates stabilization in the endocrine pancreatic indices, nutritional status, lung morphology and pulmonary function, and suggests a potentially positive impact on bone mineral metabolism and improvement in sweat chloride following one year of LUM-IVA treatment. This is a new era for CF patients in which CFTR modulators, initiated at the earliest age possible, are about to completely change the course of the disease, not only in the respiratory aspect but possibly in every system in the body that involves CFTR function. Therefore, further larger studies with a heterogeneous patient population in terms of age and disease severity, should continue investigating the effect of CFTR modulators on extra-pulmonary manifestations. This will have important implications on the need to continue other medications that now comprise the patients' pharmacological regimen.

## Supplementary Information

**Additional file 1: Supplementary Table 1.** Changes in nutritional status (BMI and Albumin) from Baseline by visit. **Supplementary Table 2.** Parameters of pulmonary exacerbations one year before and after LUM-IVA treatment.

## Data Availability

The datasets used and/or analyzed during the current study are available from the corresponding author on reasonable request.

## Abbreviations

ANC: Absolute neutrophil count
BMI: Body Mass Index
CF: Cystic fibrosis
CFBD: CF bone disease
CFQR: CF questionnaire-revised
CFRD: CF-related diabetes
CFTR: Cystic fibrosis transmembrane conductance regulator
CRP: C-reactive protein
DEXA: Dual-energy x-ray absorptiometry
FEF25-75: Forced expiratory flow between 25 and 75
FEV1: Forced expiratory volume in 1 s
FVC: Forced vital capacity
FSH: Follicle-stimulating hormone
HbA_1_C: Hemoglobin A_1_C
LH: Luteinizing hormone
LUM-IVA: Lumacaftor/Ivacaftor
OGTT: Oral glucose tolerance test
PEx: Pulmonary exacerbations
PFT: Pulmonary function tests
PI: Pancreatic insufficient
PP: Percent predicted
PTH: Parathyroid hormone
WBC: White blood cell count
